# Bilayer Biomimetic Scaffolds Loaded with Mesenchymal Stem Cell Secretomes Promote Diabetic Wound Healing

**DOI:** 10.3390/gels11110845

**Published:** 2025-10-22

**Authors:** Fangling Shen, Yiting Chen, Hongwen Li, Qi Zhang, Qixiong Ji, Linyuan Zou, Zhe Wang, Zhengyao Wu, Shengkai Yu, Hua Zhang, Qin Song

**Affiliations:** 1 College of Pharmaceutical Engineering and Biotechnology, Zhejiang Pharmaceutical University, Ningbo 315100, China; shenfangling_123@163.com (F.S.); chenyiting_115@163.com (Y.C.); lihongw@mail.zjpc.net.cn (H.L.); ji_qixiong@126.com (Q.J.); zouly@zjpc.net.cn (L.Z.); wangzhesoul@126.com (Z.W.); wuzhengyao_123@163.com (Z.W.); 2 State Key Laboratory of Radiation Medicine and Protection Medical College of Soochow University, School of Radiation Medicine and Protection, Suzhou 215123, China; qzhang2012@suda.edu.cn; 3 Research Institute of Smart Medicine and Biological Engineering, Health Science Center, Ningbo University, Ningbo 315211, China; yushengkai1996@outlook.com

**Keywords:** bilayer scaffold, stem cell secretome, anti-inflammatory, diabetic wound healing

## Abstract

Diabetic ulcers are among the most common and challenging complications of diabetes mellitus, and effective therapeutic strategies remain elusive. While stem cell secretome (SCS)-based therapy has attracted considerable attention due to its regenerative potential, its direct application is hindered by low bioavailability and rapid diffusion at the wound site. To address these limitations, we designed a bilayer bacterial cellulose–gelatin (Bi-BCG) scaffold inspired by the hierarchical structure of native skin. This scaffold features a compact bacterial cellulose (BC) upper layer with nanoscale porosity and a porous BCG lower layer with pore sizes of ~52 μm, optimized for SCS delivery. The Bi-BCG scaffold demonstrated a water vapor transmission rate of 2384 g/(m^2^·24 h) and exhibited significantly improved SCS retention capacity while maintaining high fluid absorption, outperforming monolayer BCG scaffolds. Functionally, human umbilical cord-derived mesenchymal stem cell (hUCMSCs)-derived secretomes significantly enhanced the proliferation (by up to 70.7%) and migration of skin fibroblasts under high-glucose conditions, promoted vascular endothelial cell proliferation (increasing Ki-67+ cells from 25.87% to 46.89%) and angiogenic network formation, and effectively suppressed macrophage-mediated inflammatory responses and oxidative stress. In vivo, the combination of SCSs with the Bi-BCG scaffold exhibited a clear synergistic effect, achieving a wound closure rate of 78.8% by day 10 and promoting superior structural restoration with well-organized collagen deposition, outperforming either treatment alone. These findings underscore the potential of the Bi-BCG scaffold combined with SCSs as an effective strategy for enhancing diabetic wound healing.

## 1. Introduction

Diabetic foot ulcers (DFUs) are a common and severe complication of diabetes, primarily caused by chronic inflammation, peripheral arterial disease, and peripheral neuropathy. While conventional treatments, such as wound care, antibiotics, and surgical interventions, can slow the progression of DFUs, they often fall short in promoting complete wound healing and reducing disability rates [1,2,3]. Consequently, there is an urgent need to develop more effective therapeutic strategies. Mesenchymal stem cell (MSC)-based regenerative therapies are gaining increasing attention as a promising alternative for enhancing wound healing [4,5,6]. Numerous studies indicate that while transplanted MSCs have limited long-term survival, their therapeutic effects are largely attributed to the paracrine factors they secrete [7,8,9]. The stem cell-derived secretomes (SCSs) include extracellular vesicles and a variety of bioactive molecules, including cytokines, growth factors, and chemokines. These components have been shown to significantly enhance skin cell proliferation and migration, promote angiogenesis, and modulate inflammatory responses [10,11]. Critically, emerging evidence underscores that the synergistic action of multiple growth factors within SCSs, such as VEGF and FGF, can potently activate regenerative pathways in quiescent cells, a mechanism recently highlighted in stem cell activation studies [12]. Compared to therapies based on a single growth factor, SCSs are anticipated to produce faster and more effective diabetic wound healing outcomes [13,14]. Despite these advantages, direct injection of paracrine factors into the wound site often leads to rapid degradation and a short duration of action due to their instability in vivo [15,16,17]. Therefore, developing a controllable local delivery system is essential for enhancing the retention and therapeutic efficacy of the secretome at the target site.

Various types of biomaterial scaffolds have been investigated as effective delivery platforms for the SCSs [18,19,20]. These scaffolds not only preserve the biological activity of the secretome but also function as advanced wound dressings that promote wound healing more effectively [21,22]. Among these, asymmetric scaffolds that mimic the native structure of human skin have become a research hotspot in the development of multifunctional wound dressings [23,24,25]. Natural skin is a heterogeneous tissue, consisting of a dense epidermis that acts as a barrier to prevent fluid loss and pathogen invasion, and a porous dermis that facilitates nutrient exchange and cellular activities. Inspired by this structure, asymmetric bilayer scaffolds feature a dense outer layer that provides protective shielding while regulating moisture and gas exchange. Meanwhile, the porous inner layer absorbs exudates, supports cell adhesion, migration, and proliferation, and enables the sustained release of therapeutic agents. This synergistic, biomimetic design offers significant advantages for promoting wound healing [26,27,28].

Bacterial cellulose (BC) is particularly attractive due to its three-dimensional nanofiber network structure, which closely resembles the extracellular matrix, as well as its mechanical properties that align closely with those of native skin tissue [29,30,31]. The pore size of the BC nanofiber network typically ranges from 0.05 to 5 μm, allowing it to effectively mimic the epidermis. When combined with a porous foam scaffold, BC contributes to the creation of a biomimetic asymmetric scaffold that leverages the strengths of both materials. Gelatin, a collagen-derived biomaterial, is renowned for its excellent biocompatibility, low immunogenicity, and biodegradability, making it a popular choice in tissue engineering [32,33,34]. However, gelatin-based porous scaffolds tend to absorb significant amounts of water, and the resulting swelling severely undermines their structural integrity, consequently impairing their surgical handling [35,36]. To address these challenges, we propose that incorporating BC slurry into a gelatin solution to form a composite porous scaffold may significantly enhance its mechanical strength. Additionally, integrating this composite with a bacterial cellulose hydrogel could facilitate the development of a structurally stable and mechanically robust asymmetric bilayer scaffold.

In this study, we engineered a biomimetic asymmetric bilayer scaffold comprising a dense BC hydrogel membrane functioning as the upper barrier layer. Subsequently, a composite solution of BC slurry and gelatin (denoted as BCG) was applied to the membrane’s surface and freeze-dried to produce the porous lower layer. This bilayer architecture synergistically combines the protective attributes of the dense membrane with the loading and controlled release capabilities inherent to the porous scaffold, facilitating the delivery of bioactive secretome. The conditioned medium derived from human umbilical cord-derived mesenchymal stem cells (hUCMSCs) was introduced into the bilayer BCG scaffolds (referred to as Bi-BCG). The modulatory effects of hUCMSCs-derived secretomes were systematically evaluated on skin cells, vascular endothelial cells, and macrophages, while also determining the biocompatibility of the scaffold material. Furthermore, a diabetic mouse wound model was established to explore the synergistic effect of the stem cell secretomes (SCSs) in conjunction with the biomimetic bilayer scaffold (Bi-BCG) on chronic wound healing. The results demonstrated that the biomimetic Bi-BCG scaffold loaded with hUCMSC-derived secretomes significantly enhanced the healing process by accelerating the granulation tissue formation and vascularization in diabetic wounds. This bioactive biomaterial represents a potentially translatable tissue engineering strategy and material-based solution for the treatment of diabetic wounds.

## 2. Results and Discussion

### 2.1. SCSs Promote Skin Cell Proliferation and Migration and Attenuate Oxidative Stress

**Figure 1:**
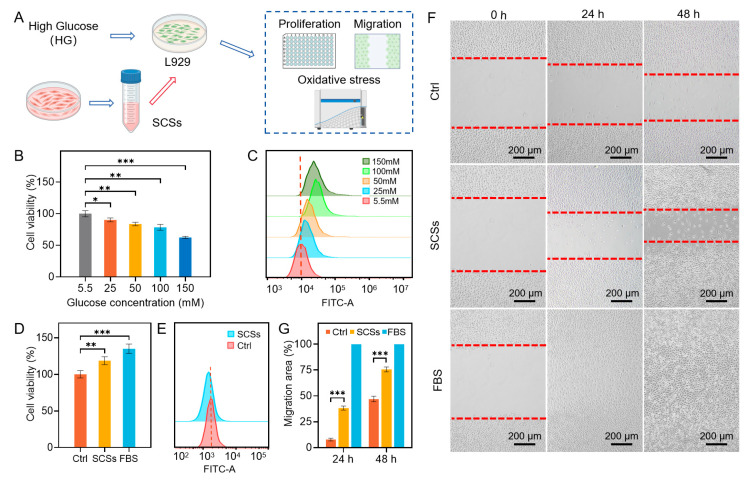
The effects of SCSs on the proliferation and migration of L929 cells impaired by high-glucose. (**A**) Schematics of collecting hUCMSC-conditioned medium used as the SCSs, establishing high-glucose (HG) impaired L929 cells, and assessment of SCSs effects on proliferation, migration, and oxidative stress. (**B**) Cell viability of L929 cells exposed to varying glucose concentrations. (**C**) Oxidative stress levels in L929 cells under varying glucose concentrations. (**D**) SCS treatment rescues L929 cell viability impaired by 25 mM HG. (**E**) SCSs alleviates oxidative stress in HG-treated L929 cells. (**F**,**G**) Scratch migration assay (representative images and quantification at 24/48 h). Statistical significance was set as * *p* < 0.05, ** *p* < 0.01, and *** *p* < 0.001.

The conditioned medium from hUCMSCs was collected after culturing for 48 h and used as the source of bioactive SCSs for diabetic wound treatment. To investigate the effects of these secretomes on fibroblast proliferation, oxidative stress, and migration, L929 fibroblasts were exposed to high glucose concentrations (25, 50, 100, and 150 mM) for 48 h to simulate the hyperglycemic microenvironment encountered in diabetic wounds (Figure 1A). As a control, we utilized stem cell culture medium without prior cell incubation in all experiments. Cell viability was assessed using the CCK-8 assay, while intracellular ROS levels were measured by flow cytometric analysis using fluorescent probe 2′,7′-dichlorodihydrofluorescein diacetate (DCFH-DA), which is widely used to detect reactive oxygen species (ROS) and enables direct measurement of intracellular ROS levels [37]. As shown in Figure 1B, the optical density values in all high-glucose groups were lower than those in the normal glucose (NG, 5.5 mM) group. Moreover, the inhibitory effect of glucose on cell proliferation demonstrated a clear concentration-dependent pattern. These results were corroborated by flow cytometric analysis of ROS levels (Figure 1C). Among the tested conditions, 25 mM glucose was selected for subsequent experiments because it not only significantly suppressed proliferation and elevated ROS but also closely mimics the physiological hyperglycemic conditions encountered in diabetic wounds, thereby providing a clinically relevant in vitro model.

The potential of SCSs to counteract the detrimental effects of high glucose on skin cells was subsequently evaluated using L929 cells. After treating L929 cells with 25 mM glucose and SCSs for 48 h, cell proliferation increased significantly by 18.6% compared to the negative control (FBS-free DMEM) (Figure 1D). Similar pro-proliferative effects were observed in normal human dermal fibroblasts (NHDFs) and human keratinocytes (HaCaTs), which exhibited increases of 70.7% and 25.7%, respectively (Figures S1A and S2A). These results demonstrate the broad beneficial influence of SCSs across different skin cell types, highlighting their potential in promoting skin regeneration in high-glucose environments.

To further assess the capacity of SCSs to reduce oxidative stress under high glucose conditions, intracellular ROS was measured via DCFH-DA fluorescence and flow cytometry. As shown in Figure 1E, L929 cells treated with SCSs alongside 25 mM glucose demonstrated significantly reduced fluorescence intensity in comparison to the high-glucose control group, indicating that SCSs substantially alleviate glucose-induced oxidative stress. Subsequently, we examined the potential of SCSs to improve cell migration, a critical process in wound healing [38,39]. Employing a scratch migration closure assay, we observed that SCSs significantly enhanced the migration of L929 cells at both 24 h and 48 h relative to FBS-free DMEM (Figure 1F). Quantitative analysis of the migration area confirmed these observations (Figure 1G). In alignment with the proliferation behavior, SCSs also promoted migration in NHDFs and HaCaTs (Figures S1B,C and S2B,C), underscoring their potential as a pro-healing therapeutic strategy for diabetic wounds.

### 2.2. SCSs Stimulate HUVEC Proliferation and Angiogenesis

**Figure 2:**
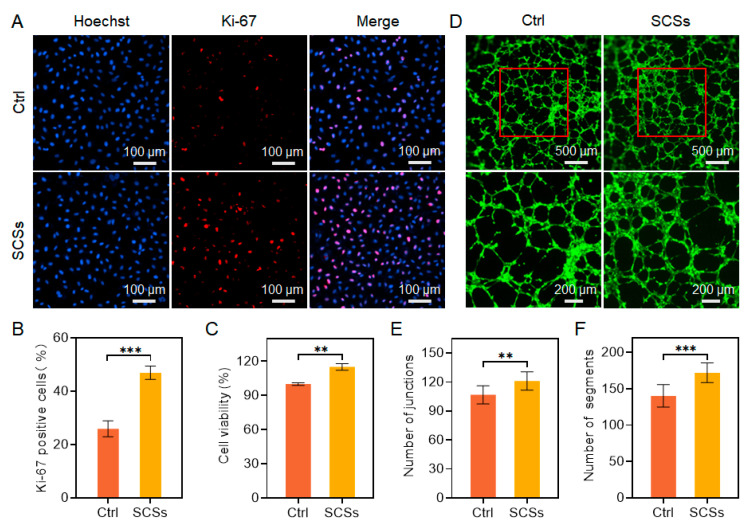
Proliferation and angiogenesis of HUVECs treated with SCSs. (**A**,**B**) Representative images of Ki-67 immunofluorescence staining and quantitative analysis of Ki-67-positive cells. (**C**) Cell viability of HUVECs measured by the CCK-8/MTS assay. (**D**) Representative images of the tube formation assay. (**E**,**F**) Quantitative analysis of total tube length and branch points. Statistical significance was set as ** *p* < 0.01, and *** *p* < 0.001.

Angiogenesis plays a crucial role in skin wound healing by facilitating the delivery of oxygen, nutrients, and growth factors to damaged tissues [40]. In diabetic conditions, however, endothelial dysfunction leads to impaired angiogenesis, resulting in poor vascularization and delayed wound closure [41]. Given that SCSs are rich in pro-angiogenic factors such as vascular endothelial growth factor (VEGF) and angiopoietins [42], we aimed to evaluate whether SCSs could enhance endothelial cell function and support angiogenic processes. To assess the effect of SCSs on endothelial proliferation, we performed immunofluorescence staining for Ki-67, a well-established marker of cell proliferation. The proportion of Ki-67-positive HUVECs significantly increased to 46.89% after treatment with SCSs, nearly doubling from 25.87% in the control group (Figure 2A,B), indicating a strong pro-proliferative effect of SCSs. This pro-proliferative effect was further corroborated by CCK-8 assays, which revealed enhanced metabolic activity following SCSs administration (Figure 2C). These results demonstrate that endothelial cell proliferation is robustly stimulated by SCSs, implicating their potential role in re-establishing vascularization in diabetic wounds.

We subsequently examined the effect of SCSs on the tube-forming capacity of HUVECs using a basement membrane extract-based tube formation assay. As shown in Figure 2D, HUVECs treated with SCSs formed extensive capillary-like networks within a 6 h period. Quantitative analysis further revealed a significant increase in both the number of junctions and segments compared to control conditions (Figure 2E,F). These results indicate that SCSs not only promote endothelial proliferation but also functionally accelerate the formation of vasculogenic structures, highlighting their therapeutic potential for improving angiogenesis in diabetic wound healing.

### 2.3. SCSs Inhibit RAW264.7 Inflammation and Oxidative Stress

**Figure 3:**
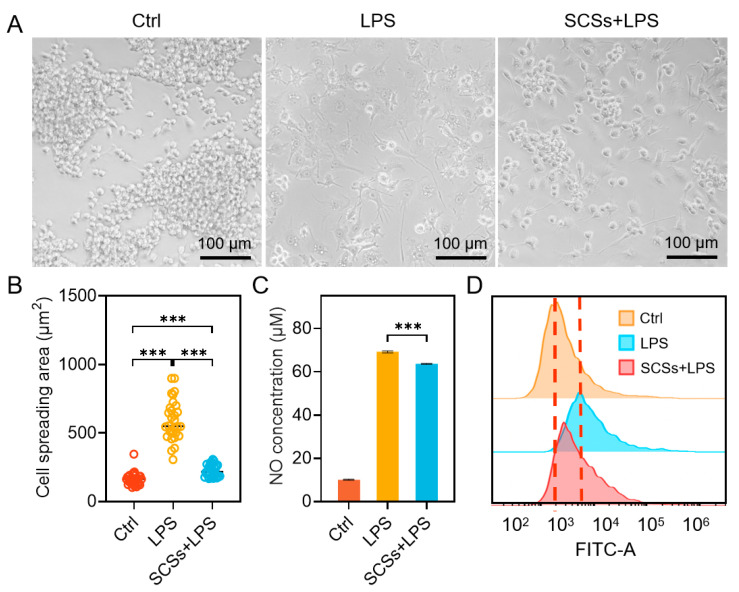
SCSs suppress inflammation and oxidative stress in RAW264.7 cells (**A**) Bright-field microscopy images of RAW264.7 cells following SCSs treatment. (**B**) Quantitative analysis of cell spreading area. (**C**) Quantitative analysis of nitrite concentration in culture supernatants from RAW264.7 cells following SCSs treatment. (**D**) Representative flow cytometry histograms of ROS levels in RAW264.7 cells following SCSs treatment. Statistical significance was set as *** *p* < 0.001.

Persistent chronic inflammation significantly impedes diabetic wound healing, necessitating the development of strategies to modulate the immune microenvironment for effective treatment [43,44]. To evaluate the potential of SCSs in regulating macrophage-driven inflammation, we established an in vitro model using LPS-stimulated RAW264.7 macrophages to mimic a pro-inflammatory (M1-like) state. Following LPS stimulation, macrophages showed a pro-inflammatory morphology characterized by a dramatic increase in cell spreading, exhibiting an area approximately 3.6-fold greater than that of unstimulated cells, along with elongated pseudopodia (Figure 3A,B). In contrast, cells treated with SCSs during LPS stimulation exhibited significantly less pronounced morphological alterations, with a spreading area only approximately 1.4-fold that of the unstimulated group (Figure 3A,B), indicating that SCSs attenuate LPS-induced cytoskeletal remodeling. We further assessed the effect of SCSs on the key inflammatory mediator nitric oxide (NO). As shown in Figure 3C, SCSs significantly suppressed LPS-induced NO production. In addition, flow cytometric analysis revealed that SCSs effectively reduced intracellular ROS levels elevated by LPS stimulation (Figure 3D). These results indicate that SCSs exert potent anti-inflammatory and antioxidant effects in inflammatory conditions.

### 2.4. Fabrication and Characterization of Bi-BCG Scaffolds

**Figure 4:**
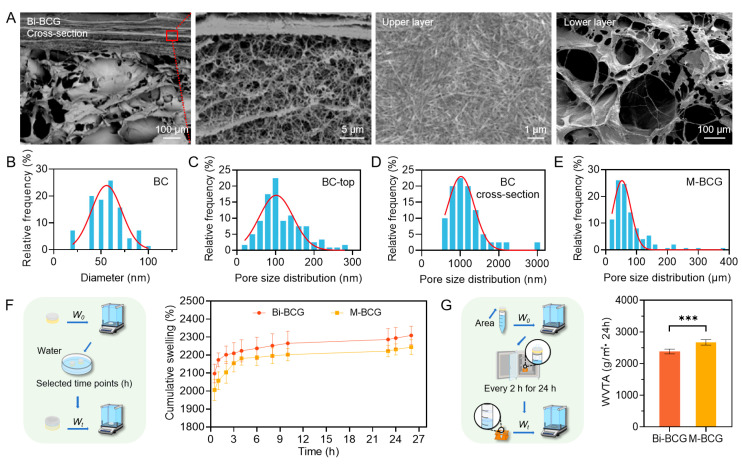
Microstructures and characterization of the Bi-BCG hydrogel scaffold. (**A**) Scanning electron microscopy (SEM) images displaying the cross-sectional view of the Bi-BCG scaffold, along with surface morphologies of the BC hydrogel upper layer and the porous lower layer. (**B**) Distribution of fiber diameters within the upper BC hydrogel. (**C**,**D**) Surface and cross-sectional pore size distributions of the upper BC hydrogel. (**E**) Pore size distributions of the porous lower layer. (**F**,**G**) Swelling behavior and water vapor transmission rate (WVTR) of the mono-layer porous scaffold (M-BCG) and the bilayer scaffold (Bi-BCG). Statistical significance was set as *** *p* < 0.001.

SCSs derived from hUCVECs significantly enhanced the proliferation and migration of cells damaged by high glucose and regulated the inflammatory response of immune cells. However, the direct injection of SCSs in vivo faces challenges such as rapid clearance, low utilization, and a lack of extracellular matrix. To achieve the efficient utilization of SCSs in vivo, it is essential to create a suitable delivery platform. Therefore, we designed an asymmetric bilayer Bi-BCG scaffold that replicates the hierarchical structure and multifunctional microenvironments of native skin, while also serving as a delivery platform for SCSs. Scanning electron microscopy (SEM) imaging revealed that the upper BC layer consists of a dense network of interwoven nanofibers with diameters ranging from 20 to 100 nm (Figure 4A,B). This upper BC layer showed a compact barrier with surface pores approximately 100 nm and side pores of around 1 μm (Figure 4A,C,D). In contrast, the lower BCG scaffold layer featured a substantially larger pore size of around 52 μm (Figure 4A,E), specifically designed to facilitate SCSs loading and support dermal tissue regeneration. This biphasic pore architecture mimics native skin stratification and provides dual functionality: the nanofibrous outer layer prevents microbial penetration, while the macroporous inner layer enables efficient incorporation and sustained release of SCSs.

We further assessed the fluid management properties of the Bi-BCG scaffold, which are crucial for creating a conducive microenvironment for SCSs delivery and healing. The bilayer hydrogel showed significantly enhanced exudate absorption and faster swelling kinetics compared to monolayer BCG (denoted as M-BCG) (Figure 4F), indicating its superior adaptability to the dynamics of wound exudate. The water vapor transmission rate (WVTR) of Bi-BCG was measured at 2384 g/(m^2^·24 h), which is lower than that of monolayer BCG (2670 g/(m^2^·24 h)) (Figure 4G) and aligns with the optimal range for wound dressings (2000–2500 g/(m^2^·24 h)) [45,46,47]. This balanced moisture control is essential for maintaining a moist interface conducive to SCSs activity and epithelial regeneration while avoiding tissue maceration or dryness, particularly advantageous in highly exudative chronic wounds.

### 2.5. Biocompatibility of Bi-BCG Scaffold

**Figure 5:**
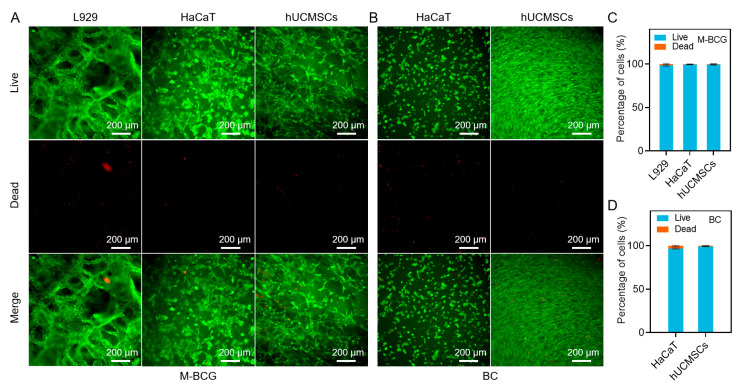
Biocompatibility of the Bi-BCG scaffold. (**A**) Live/dead fluorescence staining of L929, HaCaT, and hUCMSCs cultured on the porous BCG scaffold. (**B**) Live/dead fluorescence staining of HaCaT and hUCMSCs cultured on the BC hydrogel. (**C**,**D**) Quantitative analysis of cell viability.

To evaluate the biocompatibility of the Bi-BCG hydrogel scaffold, which is essential for its application in wound healing, we performed live/dead staining on L929 fibroblasts, HaCaT keratinocytes, and hUCMSCs cultured on both surfaces of the scaffold for 72 h. As shown in Figure 5A,B, all three cell types exhibited high viability with no observable cytotoxicity. Moreover, the cells displayed robust adhesion and characteristic spreading morphology on the hydrogel, demonstrating favorable interaction with the scaffolds. Notably, cell viability exceeded 96% for all cell types (Figure 5C,D), confirming outstanding cytocompatibility. These results strongly suggest that the Bi-BCG hydrogel provides a non-toxic, cell-supportive microenvironment conducive to adhesion, proliferation, and regeneration, affirming its potential as a safe and effective scaffold for skin tissue engineering.

The outstanding cytocompatibility and cell-supportive function of the Bi-BCG hydrogel can be systematically attributed to its integrative design, which combines favorable physicochemical properties with essential biochemical cues. The non-toxic nature of the scaffold originates from the inherent biocompatibility of its core components, bacterial cellulose and gelatin, both well-established biomaterials. The macroporous structure of the BCG lower layer (~52 μm) provides a critical three-dimensional framework that supports cell infiltration, nutrient diffusion, and tissue integration—fundamental processes for proliferation and regeneration. Most importantly, the incorporation of gelatin introduces native RGD-like peptide sequences, which are essential cell-adhesion motifs. These biochemical cues directly promote integrin-mediated cell attachment and spreading, as clearly observed in our results (Figure 5A,B), representing the crucial initiating event for subsequent cellular processes. Furthermore, the scaffold’s optimal fluid management capacity works in concert with its porous structure to maintain a moist, hydrated microenvironment at the cell–scaffold interface, which prevents cellular desiccation and facilitates efficient gas exchange—collectively supporting high metabolic activity and the robust viability (>96%) documented across all tested cell types.

### 2.6. Accelerating Diabetic Wounds Healing with Bi-BCG Containing SCSs

**Figure 6:**
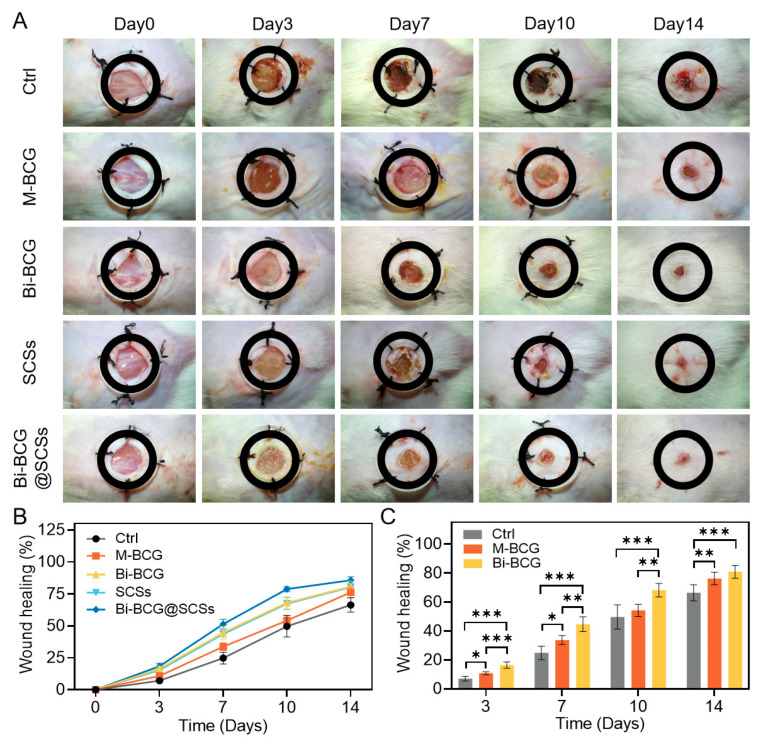
Effects of Bi-BCG hydrogel combined with SCSs on diabetic wound healing. (**A**) Representative wound images from different treatment groups at days 0, 3, 7, 10, and 14. (**B**,**C**) Quantitative analysis of wound closure rates. Ctrl: control group; M-BCG: mono-layer porous scaffold group; Bi-BCG: bilayer scaffold group; SCSs: stem cell secretomes; Bi-BCG@SCSs: bilayer scaffold combined with stem cell secretomes. Statistical significance was set as * *p* < 0.05, ** *p* < 0.01, and *** *p* < 0.001.

Diabetic complications often lead to delayed wound healing and the development of chronic ulcers, posing substantial challenges to clinical management [2]. To assess the therapeutic potential of the Bi-BCG@SCSs composite in diabetic wound repair, we established a streptozotocin (STZ)-induced diabetic ICR mouse model. Diabetes was confirmed by sustained random blood glucose levels exceeding 16.7 mmol/L for three consecutive days [48], with progressive hyperglycemia documented over time (Table S1). A full-thickness dorsal wound measuring 8 mm diameter was created, and the animals were divided into five treatment groups: Bi-BCG@SCSs, BCG scaffold, Bi-BCG scaffold, SCSs alone, and DMEM control. The macroscopic wound closure over a 14-day period was carefully evaluated. Representative images of the wounds were captured on days 0, 3, 7, 10, and 14 (Figure 6A). By day 10, the differences in healing among the groups were most pronounced. The Bi-BCG@SCSs group exhibited the fastest healing rate, achieving a closure rate of 78.8 ± 0.9%, which was significantly higher than those in the Bi-BCG scaffold (68.2 ± 2.1%), SCSs (67.4 ± 2.1%), BCG scaffold (54.3 ± 1.9%), and control (49.8 ± 3.8%) groups (Figure 6B). Notably, the Bi-BCG scaffold alone also outperformed the BCG group, underscoring the advantages of the bilayered design in enhancing wound healing.

**Figure 7:**
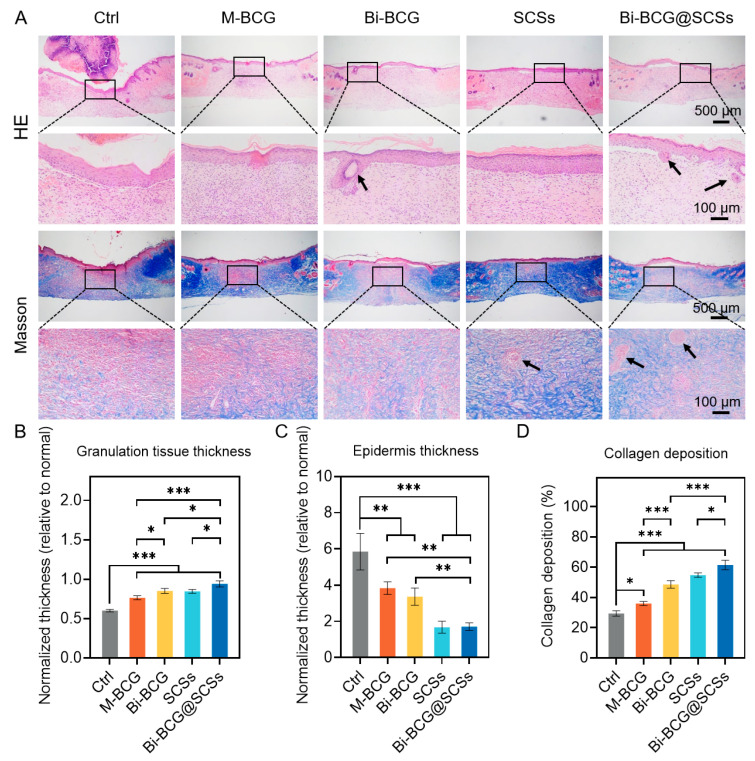
Pathological sections of wound tissue at day 14 post-treatment. (**A**) Representative H&E and Masson staining of different treatment groups; black arrows indicate regenerated skin appendages. (**B**,**C**) Normalized thickness of granulation tissue and epidermis, with thickness values of wound tissues normalized to those of normal skin (set as 1); (**D**) Quantification of collagen fiber content. Ctrl: control group; M-BCG: mono-layer porous scaffold group; Bi-BCG: bilayer scaffold group; SCSs: stem cell secretomes; Bi-BCG@SCSs: bilayer scaffold combined with stem cell secretomes. Statistical significance was set as * *p* < 0.05, ** *p* < 0.01, and *** *p* < 0.001.

Histological analysis conducted on day 14 provided further insight into the quality of wound regeneration. H&E staining revealed the presence of well-structured granulation tissue and newly formed hair follicles in the Bi-BCG@SCSs group, whereas the control group showed incomplete epithelialization (Figure 7A). Quantitative measurements indicated that the Bi-BCG@SCSs group exhibited the greatest normalized thickness of granulation tissue, indicating enhanced wound bed formation. Moreover, the epidermal thickness in the Bi-BCG@SCSs closely approximated that of native skin, demonstrating superior structural restoration (Figure 7B,C). Masson’s trichrome staining demonstrated dense and well-organized collagen fibers in the Bi-BCG@SCSs-treated wounds, contrasting with the sparse, disordered collagen and persistent scab observed in controls (Figure 7D). Intermediate collagen deposition levels were noted in the SCSs and Bi-BCG scaffold groups. The enhancement in wound healing associated with Bi-BCG@SCSs can be attributed to the sustained release of bioactive factors such as VEGF, FGF, and TGF-β from the secretomes, which promote angiogenesis, fibroblast proliferation, and collagen remodeling [49,50,51]. Concurrently, the bilayered hydrogel acts as a biomimetic scaffold that supports cell infiltration, maintains moisture balance, and protects the wound bed [25,52]. Collectively, these characteristics foster a regenerative microenvironment conducive to rapid and ordered tissue repair.

These histological outcomes demonstrate the strong therapeutic efficacy of the SCSs delivered via the Bi-BCG scaffold, underscoring its potential as a localized treatment for diabetic wound healing. However, several limitations of this study should be acknowledged. Due to the nature of the intervention, blinding during experimental procedures was not feasible, which may have introduced performance bias, although outcome assessors and data analysts were blinded to mitigate this risk. In addition, the exclusive use of male ICR mice limits the generalizability of the findings to other strains or sexes, and the acutely induced diabetic model does not fully replicate the chronic, multifactorial progression of human diabetes.

Further clinical translation of SCSs based therapy also faces challenges, particularly given the inherent complexity of the secretome, which comprises a diverse mixture of growth factors, cytokines, and extracellular vesicles. This complexity necessitates rigorous quality control to ensure batch to batch consistency and safety. In this study, we employed standardized culture conditions and functional potency assays to qualify SCSs batches as an initial step toward standardization. Combining SCSs with the well-defined Bi-BCG scaffold creates a controlled, localized delivery system that reduces off-target effects and may simplify the regulatory pathway for this complex biologic.

Future work will focus on validating these findings in more diverse animal models and further investigating the translational potential of this strategy. Detailed proteomic and vesicle profiling will be conducted to establish definitive critical quality attributes that correlate with in vivo efficacy, thereby paving the way for reproducible and com-pliant clinical manufacturing.

## 3. Conclusions

In summary, this study developed a bilayer bacterial cellulose–gelatin (Bi-BCG) scaffold that provided a reliable delivery platform for stem cell secretomes (SCSs), effectively overcoming critical challenges such as poor retention and rapid degradation of bioactive factors. The biomimetic architecture of the scaffold not only replicated the layered organization of native skin but also provided a protective and moist microenvironment conducive to healing. Functionally, SCSs derived from hUCMSCs significantly enhanced fibroblast and endothelial cell proliferation, attenuated inflammatory responses, and reduced oxidative stress. The synergistic combination of Bi-BCG and SCSs markedly accelerated wound closure, improved granulation tissue formation, and enhanced vascularization in a diabetic mouse model, outperforming either component alone. While future studies are needed to validate these findings in more diverse models and to establish standardized quality control protocols for clinical translation, the Bi-BCG@SCS combination shows great promise for the treatment of refractory diabetic wounds, paving the way for future clinical applications and research in regenerative medicine.

## 4. Materials and Methods

### 4.1. Materials

Male ICR mice (6-week-old) were purchased from Laboratory Animal Center of Hangzhou Medical College (Hangzhou, China). Cell Counting Kit-8 (CCK-8), Griess Reagent System, Reactive Oxygen Species Assay Kit, Hematoxylin and Eosin (H&E) Staining Kit, Masson’s Trichrome Staining Kit, Calcein-AM/PI and Hoechst 33342 were purchased from Beyotime Biotechnology (Shanghai, China). Streptozotocin (STZ) was purchased from Sigma-Aldrich (St. Louis, MO, USA). Fetal bovine serum (FBS) was obtained from CellMax (Beijing, China). Dulbecco’s Modified Eagle Medium (DMEM), Dulbecco′s Modified Eagle′s Medium-low glucose (DMEM-LG), and Minimum Essential Medium α (MEMα) were purchased from Gibco (Grand Island, NY, USA). Ki-67 polyclonal antibody and anti-rabbit secondary antibody were obtained from Proteintech (Wuhan, China). All other chemical reagents were of analytical grade.

### 4.2. Cell Culture and Collection of hUCMSC-Conditioned Medium (CM)

The experimental protocol was approved by the Ethics Committee of Soochow University (20230314). The isolation procedure was adapted from a previously described method with minor modifications [53,54]. Briefly, the umbilical cord was dissected to remove the umbilical artery and vein, and the remaining tissue was minced into small fragments. These fragments were cultured in α-MEM medium supplemented with 10% fetal bovine serum (FBS) at 37 °C in a humidified atmosphere of 5% CO_2_. Upon reaching confluence, the cells were passaged using enzymatic digestion.

For the preparation of conditioned medium, hUCMSCs at passages 5 to 10 were used. When UCMSCs reached approximately 80% confluence, the cells were washed three times with phosphate-buffered saline (PBS). The medium was then replaced with DMEM-LG, and cells were incubated under standard conditions (37 °C, 5% CO_2_) for 48 h. The supernatant was collected, centrifuged at 400× *g* for 10 min, and filtered through a 0.22 μm membrane. The conditioned medium (CM) as a source of Stem cell secretomes (SCSs) was aliquoted and stored at −80 °C until use.

The L929, HaCaT, NHDF, RAW264.7, and HUVEC cell lines were purchased from FuHeng Biology (Catalog No. FH0534), Kunming Cell Bank of the Chinese Academy of Sciences (Catalog No. 5301HUM-KCB04045YJ), BeNa Culture Collection (BNCC; Catalog No. BNCC358600 and BNCC354753, respectively), and Wuhan PriCells Life Technologies Co., Ltd. (Catalog No. CL-0675L929).

All cells were cultured in DMEM supplemented with 10% FBS at 37 °C in a humidified 5% CO_2_ incubator. The L929, HaCaT, NHDF, and HUVEC cells were passaged using 0.25% trypsin-EDTA, while RAW264.7 macrophages were passaged by gentle pipetting.

### 4.3. Cell Viability Assay

To establish a hyperglycemic model, L929 cells were cultured in media containing different concentrations of glucose (25, 50, 100, and 150 mM) for 48 h. Cell viability was assessed using the CCK-8 assay, and a glucose concentration that closely mimics physiological hyperglycemic conditions and significantly inhibits L929 proliferation was selected for subsequent experiments.

To evaluate the effects of SCSs, L929 cells were seeded in 96-well plates at a density of 5 × 10^4^ cells/mL and cultured for 48 h in DMEM containing 25 mM glucose. The medium was then replaced with hUCMSC-conditioned medium (also containing 25 mM glucose) for an additional 48 h. DMEM with 25 mM glucose served as the negative control and the same medium supplemented with 10% FBS as the positive control. After treatment, the medium was replaced with DMEM containing 10% CCK-8 reagent, and the cells were incubated for 1 h in the dark. Optical density was measured at 450 nm using a microplate reader (SpectraMax M2, Molecular Devices, San Jose, CA, USA).

### 4.4. Reactive Oxygen Species (ROS) Detection

To investigate the effects of high glucose on oxidative stress, L929 cells were cultured in media containing different glucose concentrations (25, 50, 100, and 150 mM) for 48 h. Intracellular ROS production was assessed using the DCFH-DA fluorescent probe. The glucose concentration that most closely mimicked physiological hyperglycemic conditions and significantly induced ROS production was selected for subsequent experiments.

According to the manufacturer’s instructions, L929 cells were seeded in 12-well plates at a density of 5 × 10^4^ cells/mL and cultured for 48 h in DMEM supplemented with 25 mM glucose. The medium was then replaced with hUCMSC-conditioned medium (also containing 25 mM glucose) for another 48 h. DMEM with 25 mM glucose served as control. After incubation, the cells were treated with 10 μM DCFH-DA for 20 min at 37 °C under 5% CO_2_. Fluorescence intensity was measured by flow cytometry (CytoFLEX, Beckman Coulter, Brea, CA, USA) with excitation at 488 nm and emission at 525 nm.

### 4.5. Scratch Assay

A scratch assay was performed to assess cell migration. L929 cells were seeded in 12-well plates at 5 × 10^4^ cells/mL and cultured until 100% confluence. The cells were then assigned to three treatment groups, all containing 25 mM glucose: negative control (DMEM), hUCMSC-conditioned medium, and positive control (DMEM supplemented with 10% FBS). Parallel scratches were created in each well using a sterile 200 μL pipette tip.

Cells were incubated with the indicated treatments for 0, 24, and 48 h at 37 °C under 5% CO_2_. Scratch areas were imaged using a phase-contrast microscope (BDS400, CNOPTEC, Chongqing, China). Scratch widths at baseline (*A*_0_) and at different time points (*A_t_*) were quantified using ImageJ software (version 1.54p, National Institutes of Health, Bethesda, MD, USA). The migration ratio was calculated according to the following formula:

(1) $$\mathrm{Migration}\;\mathrm{ratio}\;(\%)\;=\;\frac{A_{t}-A_{0}}{A_{0}}\;\times \;100$$

### 4.6. Immunofluorescence Staining of Ki-67

HUVECs were seeded in 96-well plates at a density of 7 × 10^4^ cells/mL and cultured for 24 h, followed by replacement with hUCMSC-conditioned medium for another 24 h. DMEM-treated cells served as the control. Cells were fixed with 4% paraformaldehyde for 20 min, permeabilized with 0.1% Triton X-100 for 5 min, and blocked with 5% bovine serum albumin (BSA) for 1 h at room temperature. Subsequently, cells were incubated overnight at 4 °C with primary antibodies against Ki-67, followed by fluorophore-conjugated secondary antibodies for 30 min at room temperature. Nuclei were counterstained with Hoechst 33342. Images were acquired using a fluorescence microscope (ECLIPSE TS2R-FL, Nikon, Tokyo, Japan).

### 4.7. Endothelial Tube Formation Assay

To evaluate angiogenic potential, Matrigel was thawed overnight at 4 °C and added to pre-cooled 96-well plates, followed by incubation at 37 °C for 30 min to allow solidification. HUVECs (3 × 10^4^ cells/well) were seeded onto the Matrigel and treated under different conditions for 3–10 h. Tube formation was assessed using an inverted microscope (ECLIPSE TS2R-FL, Nikon, Japan). The number of junctions and segments was quantified with ImageJ software (version 1.54p, NIH, Bethesda, MD, USA) using the Angiogenesis Analyzer plugin.

### 4.8. Nitric Oxide (NO) Production Assay

RAW264.7 cells were seeded in 96-well plates at 1 × 10^6^ cells/mL and cultured for 24 h, followed by replacement with hUCMSC-conditioned medium in the presence of 100 ng/mL LPS for another 24 h. Cells treated with DMEM without LPS served as the negative control, while those receiving DMEM with 100 ng/mL LPS served as the positive control. The culture supernatants were collected, and NO production was determined using the Griess Reagent System following the manufacturer’s instructions. Absorbance was measured at 540 nm with a microplate reader (SpectraMax M2, Molecular Devices, San Jose, CA, USA).

### 4.9. Preparation of Bilayer Scaffolds

Bacterial cellulose (BC) hydrogel was prepared following a previously established method with minor modifications [55]. Briefly, BC hydrogel was prepared via static fermentation at 30 °C using Acetobacter xylinum NUST4.2, followed by treatment with 0.1 M sodium hydroxide solution at 80 °C for 2 h. The samples were thoroughly rinsed with double-distilled water and PBS several times to remove bacteria and residual alkali. After autoclaving, BC hydrogels were stored at 4 °C until use.

The bilayer hydrogel scaffold (BC-GEL) was prepared as follows: BC hydrogel was cut to fit the mold and placed at the bottom. A 3% gelatin solution containing 0.6% glycerol was mixed with BC slurry (1:1, *v*/*v*) and cast onto the BC hydrogel. The samples were freeze-dried (SCIENTZ-12, Scientz, Ningbo, China) and crosslinked at 140 °C to obtain bilayer hydrogels. Before use, the scaffolds were sterilized by UV irradiation.

Porous hydrogels were prepared by directly casting the gelatin/BC slurry mixture into molds, followed by the same procedures.

### 4.10. SEM Characterization of Hydrogel Structure

The microstructure of the hydrogels was observed using a scanning electron microscope (Pharos G2, Thermo Fisher Scientific, Waltham, MA, USA). Samples were freeze-dried, sputter-coated with gold, and imaged at an accelerating voltage of 4 kV. Pore size and fiber diameter were analyzed using ImageJ software (NIH, Bethesda, MD, USA).

### 4.11. Swelling Behavior

The swelling behavior was determined using a method consistent with the previously described procedure, with minor modifications [56]. Briefly, lyophilized hydrogel samples were weighed (*W*_0_) and immersed in ultrapure water at 37 °C. At predetermined intervals, swollen samples were removed, blotted to remove surface water, and weighed (*W_t_*). The swelling ratio (SR) was calculated as:

(2) $$\mathrm{SR}(\%)\;=\;\frac{\;W_{t}-W_{0}}{W_{0}}\;\times \;100$$

### 4.12. Water Vapor Transmission Rate (WVTR) Determination

Hydrogel samples were cut into disks and sealed across the mouths of 15 mL cylindrical cups containing 10 mL ultrapure water with parafilm. The cups were placed in an incubator at 37 °C and 50% relative humidity. The assemblies were weighed every 2 h for 24 h, with six replicates per group. WVTR was calculated as:

(3) $$\mathrm{WVTR}\;=\;\frac{∆W}{A\;\times \;t}$$

where Δ*W* is the weight loss (g), *A* is the exposed area (cm^2^), and *t* is the time (24 h).

### 4.13. Cytocompatibility of Bi-BCG Scaffolds

To evaluate cytocompatibility, cells were seeded on both sides of the BC-GEL bilayer hydrogel at a density of 5 × 10^4^ cells/mL and cultured at 37 °C with 5% CO_2_ for 48 h. Cell viability was evaluated by Calcein-AM/PI double staining. After PBS washing, samples were observed under a fluorescence microscope (ECLIPSE TS2R-FL, Nikon, Japan). Live cells exhibited green fluorescence, whereas nuclei of dead cells showed red fluorescence.

### 4.14. Diabetic Wound Model

Healthy male ICR mice (6–8 weeks) were used. All procedures were approved by the Institutional Animal Care and Use Committee (IACUC) of Zhejiang Chinese Medical University (20220819). Diabetes was induced by intraperitoneal injection of streptozotocin (STZ, 50 mg/kg) for five consecutive days. One week later, blood glucose was measured, and mice exhibiting random blood glucose levels > 16.7 mM for three consecutive days were considered diabetic. After anesthesia, full-thickness excisional wounds (8 mm in diameter) were created on the dorsal skin using a biopsy punch.

### 4.15. In Vivo Wound Healing Evaluation

Diabetic mice were randomly divided into five groups: Ctrl: DMEM control; M-BCG: mono-layer porous scaffolds; Bi-BCG: bilayer scaffolds; SCSs: stem cell secretomes; Bi-BCG@SCSs: bilayer scaffold combined with stem cell secretomes. Each group contained at least six mice. Treatments were applied every three days. Wound healing progression was monitored for 14 days. Photographs were taken on days 0, 3, 7, 10, and 14. Wound areas were measured using ImageJ software (NIH, USA), and wound closure rate was calculated as:

(4) $$\mathrm{Wound}\;\mathrm{closure}\;\mathrm{rate}\;(\%)\;=\frac{S_{0}-S_{t}}{S_{0}}\;\times \;100$$

where *S*_0_ is the wound area on day 0, and *S_t_* is the wound area at time *t*.

### 4.16. Histological Analysis

On day 14, regenerated skin tissues were harvested, fixed in 4% paraformaldehyde, dehydrated in graded ethanol and xylene, embedded in paraffin, and sectioned at 5 μm using a microtome (KD-2268, Kedi, Jinhua, China). Sections were stained with hematoxylin and eosin (H&E) and Masson’s trichrome according to the manufacturer’s protocol. All images were obtained with an optical microscope (BK6000, CNOPTEC, China). Quantitative analysis was performed using ImageJ software (NIH, USA), and three randomly selected high-magnification fields from each section were analyzed.

### 4.17. Statistical Analysis

Data are expressed as mean ± standard deviation (mean ± SD) from at least three independent experiments. Statistical analysis was performed using one-way ANOVA with Tukey’s post hoc test (GraphPad Prism 9.5.1, GraphPad Software, San Diego, CA, USA). Statistical significance was set as * *p* < 0.05, ** *p* < 0.01, and *** *p* < 0.001.

## Data Availability

All data that support the findings of this study are included within the article and Supplementary Files.

## Supplementary Materials

The following supporting information can be downloaded at: https://www.mdpi.com/article/10.3390/gels11110845/s1, Figure S1: SCSs promote the proliferation and migration of NHDFs. (A) Cell viability quantified by CCK-8 assay. (B) Quantitative analysis of scratch closure after 48 h. (C) Representative scratch wound images at 48 h; Figure S2: SCSs promote the proliferation and migration of HaCaTs. (A) Cell viability quantified by CCK-8 assay. (B) Quantitative analysis of scratch closure after 48 h. (C) Representative scratch wound images at 48 h. Table S1: Random Blood Glucose in STZ-Induced Diabetic Mice at 2 and 7 Weeks (mmol/L).
