# Supplementary material for: Bilayer Biomimetic Scaffolds Loaded with Mesenchymal Stem Cell Secretomes Promote Diabetic Wound Healing
Source: Gels. 2025 Oct 22;11(11):845. doi: 10.3390/gels11110845 (PMC12652527; doi:10.3390/gels11110845)
Supplement: Supplementary file 1 [file gels-11-00845-s001.zip › gels-3908149-supplementary.pdf]

Bilayer Biomimetic Scaffolds Loaded with Mesenchymal Stem Cell Secretomes Promote Diabetic Wound Healing

Fangling Shen [1,†], Yiting Chen [1,†], Hongwen Li [1], Qi Zhang [2], Qixiong Ji [1], Linyuan Zou [1], Zhe Wang [1], Zhengyao Wu [1], Shengkai Yu [3], Hua Zhang [3,*] and Qin Song [1,*]

[1] College of Pharmaceutical Engineering and Biotechnology, Zhejiang Pharmaceutical University, Ningbo 315100, China; shenfangling_123@163.com (F.S.); chenyiting_115@163.com (Y.C.); lihongw@mail.zjpc.net.cn (H.L.); ji_qixiong@126.com (Q.J.); zouly@zjpc.net.cn (L.Z.); wangzhesoul@126.com (Z.W.); wuzhengyao_123@163.com (Z.W.)

[2] School of Radiation Medicine and Protection, State Key Laboratory of Radiation Medicine and Protection Medical College of Soochow University, Suzhou 215123, China; qzhang2012@suda.edu.cn

[3] Research Institute of Smart Medicine and Biological Engineering, Health Science Center, Ningbo University, Ningbo 315211, China; yushengkai1996@outlook.com

* Correspondence: zhanghua@nbu.edu.cn (H.Z.); songq@zjpu.edu.cn (Q.S.)

† These authors contributed equally to this work.

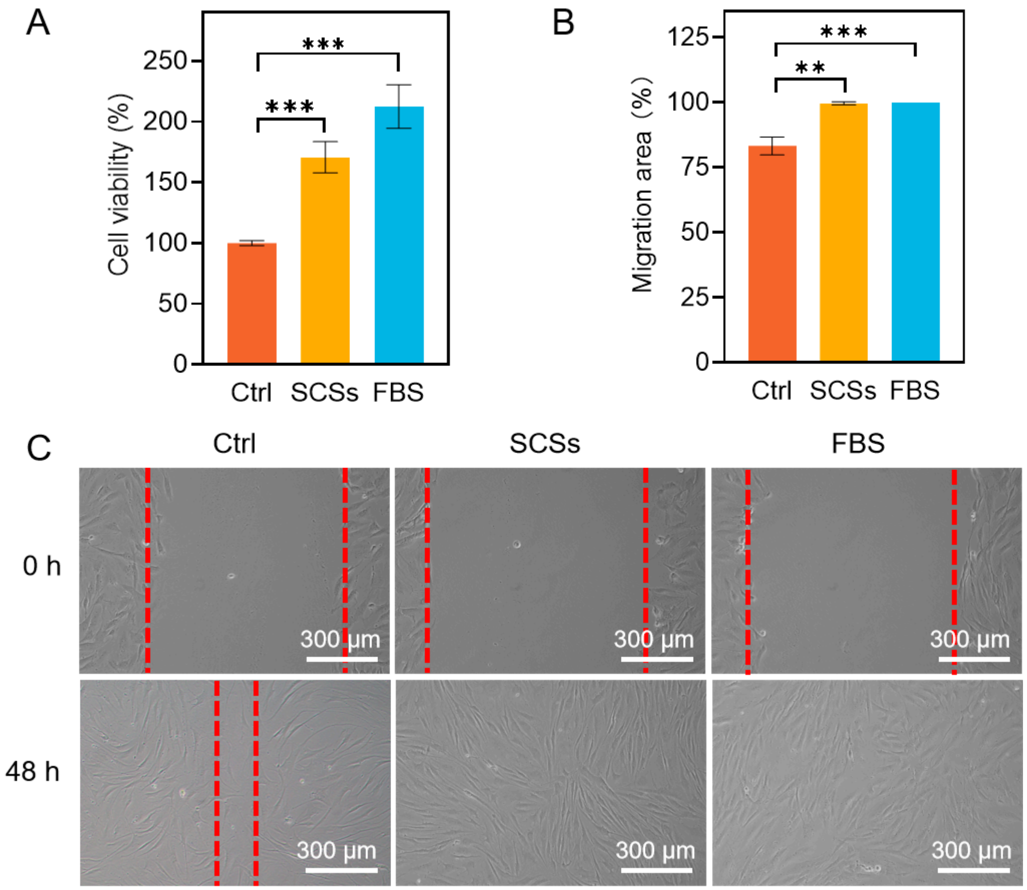

Figure S1 SCSs promote the proliferation and migration of NHDFs. (A) Cell viability quantified by CCK-8 assay. (B) Quantitative analysis of scratch closure after 48 h. (C) Representative scratch wound images at 48 h.

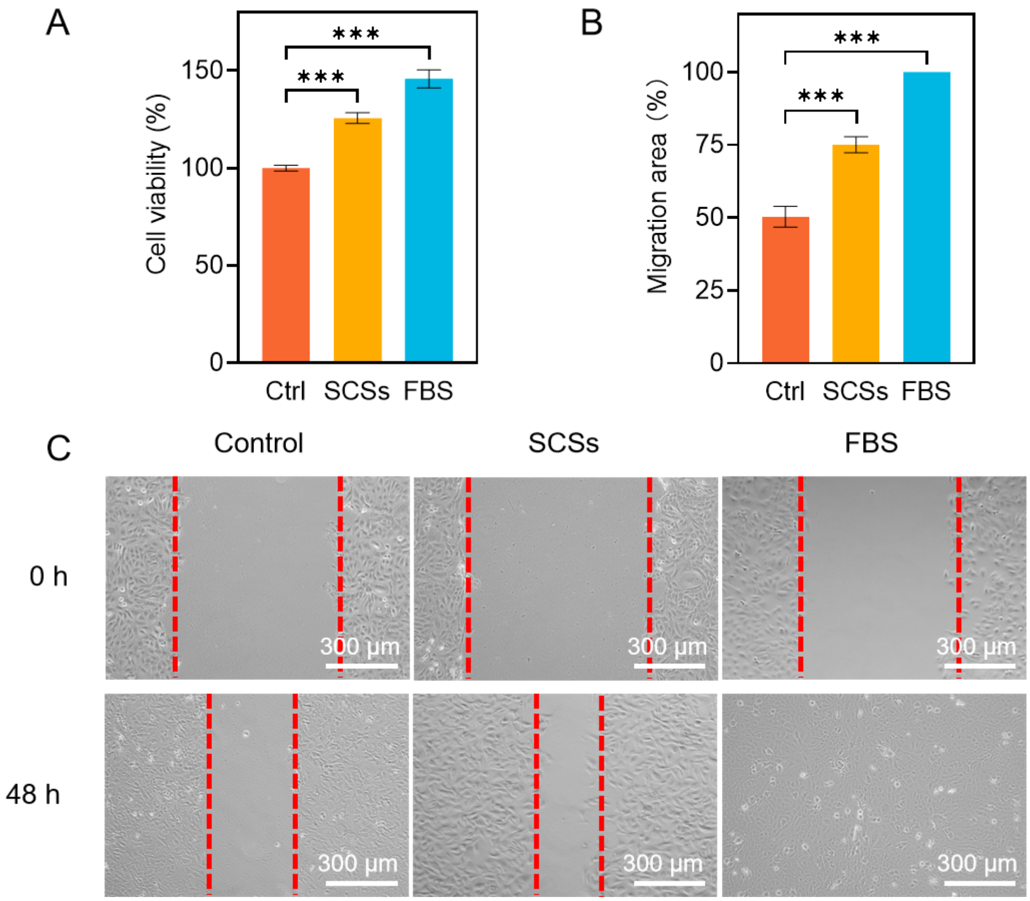

Figure S2 SCSs promote the proliferation and migration of HaCaTs. (A) Cell viability quantified by CCK-8 assay. (B) Quantitative analysis of scratch closure after 48 h. (C) Representative scratch wound images at 48 h.

Table S1 Random Blood Glucose in STZ-Induced Diabetic Mice at 2 and 7 Weeks (mmol/L)

Time point \ Mouse ID	1	2	3	4	5	6	7	8	9	10	11	12
Week 2	HI	30.7	23.7	HI	31.4	31.3	16.5	29.4	29.3	32.6	32.0	26.0
Week 7	HI	HI	32.9	HI	HI	HI	25.9	HI	HI	HI	HI	HI

Note: "HI" denotes blood glucose > **36.0 mmol/L**.
